# The Cell Cycle Regulated Transcriptome of *Trypanosoma brucei*

**DOI:** 10.1371/journal.pone.0018425

**Published:** 2011-03-31

**Authors:** Stuart K. Archer, Diana Inchaustegui, Rafael Queiroz, Christine Clayton

**Affiliations:** 1 Zentrum für Molekulare Biologie Heidelberg, DKFZ-ZMBH Allianz, Heidelberg, Germany; 2 Deutsches Krebsforschungszentrum, DKFZ-ZMBH Allianz, Heidelberg, Germany; The Rockefeller University, United States of America

## Abstract

Progression of the eukaryotic cell cycle requires the regulation of hundreds of genes to ensure that they are expressed at the required times. Integral to cell cycle progression in yeast and animal cells are temporally controlled, progressive waves of transcription mediated by cell cycle-regulated transcription factors. However, in the kinetoplastids, a group of early-branching eukaryotes including many important pathogens, transcriptional regulation is almost completely absent, raising questions about the extent of cell-cycle regulation in these organisms and the mechanisms whereby regulation is achieved. Here, we analyse gene expression over the *Trypanosoma brucei* cell cycle, measuring changes in mRNA abundance on a transcriptome-wide scale. We developed a “double-cut” elutriation procedure to select unperturbed, highly synchronous cell populations from log-phase cultures, and compared this to synchronization by starvation. Transcriptome profiling over the cell cycle revealed the regulation of at least 430 genes. While only a minority were homologous to known cell cycle regulated transcripts in yeast or human, their functions correlated with the cellular processes occurring at the time of peak expression. We searched for potential target sites of RNA-binding proteins in these transcripts, which might earmark them for selective degradation or stabilization. Over-represented sequence motifs were found in several co-regulated transcript groups and were conserved in other kinetoplastids. Furthermore, we found evidence for cell-cycle regulation of a flagellar protein regulon with a highly conserved sequence motif, bearing similarity to consensus PUF-protein binding motifs. RNA sequence motifs that are functional in cell-cycle regulation were more widespread than previously expected and conserved within kinetoplastids. These findings highlight the central importance of post-transcriptional regulation in the proliferation of parasitic kinetoplastids.

## Introduction

In the eukaryotic cell division cycle, many proteins involved in the replication of the cell and its components are specifically expressed exactly when required, ensuring tight control over replicative processes and increasing cellular efficiency. Regulation at the level of transcription has been thoroughly documented: for example, in yeast, at least nine transcription factors central to cell-cycle regulation operate in a network to control the expression of each other and of downstream effectors of cell cycle progression [1]. Downstream targets of these transcription factors include cyclins, DNA replication proteins and structural proteins such as histones. The consequences of breakdown in cell-cycle related transcriptional regulation can be severe [2]. Post-transcriptional regulatory mechanisms such as mRNA and protein degradation also play a part, for example histone mRNAs are rapidly degraded after S-phase when they are no longer required [3].

The kinetoplastids are an early-branching group of unicellular eukaryotes including several important parasitic pathogens of humans and animals. Their life-cycles involve alternation between two very different hosts, typically vertebrates and biting insects, each of which represent considerable, but very different, challenges to the parasites' survival. During parasite adaptation, the cell shape can change from long, spindle-shaped cells with flagellar-driven motility to almost spherical, immotile cells, and there are dramatic changes in metabolism and cell surface macromolecules. *Trypanosoma brucei*, the causative agent of African Sleeping Sickness (trypanosomiasis), differentiates through at least seven distinct cell types as it progresses through its life cycle, passing from mammalian hosts to the Tsetse fly vector [4]. Unusually, despite the clear need for gene regulation, kinetoplastids show near-complete absence of transcriptional control of gene expression [5]. Genes are arranged in large unidirectional arrays which are transcribed in a polycistronic fashion and subsequently cut and processed into individual, mature mRNAs. Thus it is only during and after the mRNA processing step that individual control of gene expression is possible. Nevertheless, post-transcriptional control, often involving differential rates of RNA degradation, allows rapid changes in protein levels to occur [5], [6], [7], [8]. Kinetoplastids are thus excellent models for post-transcriptional control of gene expression in eukaryotes.

Kinetoplastid parasites undergo cell division in a characteristically well-ordered way. Unlike in many other eukaryotes, subcellular structures and organelles such as the mitochondria, ER, Golgi and flagellum are present in a single copy in G1-phase cells and are replicated at defined times during the cell division cycle [9], [10], [11]. Several groups of cell-cycle regulated transcripts have been identified. The best understood is a group of genes encoding proteins required for DNA replication, e.g. thymidylate synthases, topoisomerases and DNA ligases. Originally investigated in the related kinetoplastid *Crithidia fasciculata*, these transcripts peak in abundance in early S-phase, and their regulation is dependent on the presence of one or several octameric motifs with consensus sequence [CAUAGAAG] in the untranslated regions [12]. This motif is recognized by the Cycling Sequence Binding Proteins CSBPA and CSBPB [13] and also CSBP II [14]. CSBP II consists of several proteins, some of which are differentially phosphorylated over the cell cycle [15], suggesting a likely mechanism for cell-cycle coupled transcript regulation. We previously identified a small group of mRNAs that are associated with the PUF-domain protein PUF9, and whose levels peak in late S-phase/early G2 [16]. For this group, a 3′-UTR motif with a consensus sequence [UUGUACC] seems to be necessary but not sufficient to confer regulation. Furthermore, histone mRNAs are regulated in the cell cycle [17], but the regulatory mechanism responsible has not been characterized.

So far, investigation of the kinetoplastid cell cycle has involved synchronization of cells by starvation [18] or drug-mediated inhibition of DNA synthesis [19], [20]. Here, we developed a novel procedure for the selection of highly synchronous, unperturbed *T. brucei* cells, which we used to identify regulated transcripts over the cell cycle on a transcriptome-wide scale. We cross-validated against starvation-synchronized cells and identified mRNAs that were regulated during the cell cycle: 55 peaking in early G1, 273 in late G1; 98 in S-phase and 120 in G2 phase. Genes functioning in several processes such as DNA metabolism or flagellar formation showed expression peaks at distinctive times in the cell cycle, correlating to a time just prior to the peak demand for the encoded proteins. For several groups of co-regulated transcripts, potential protein binding sites in the untranslated regions were found, which were conserved in different kinetoplastids.

## Results

### Expression profiling of starvation-synchronized cells

**Figure 1:**
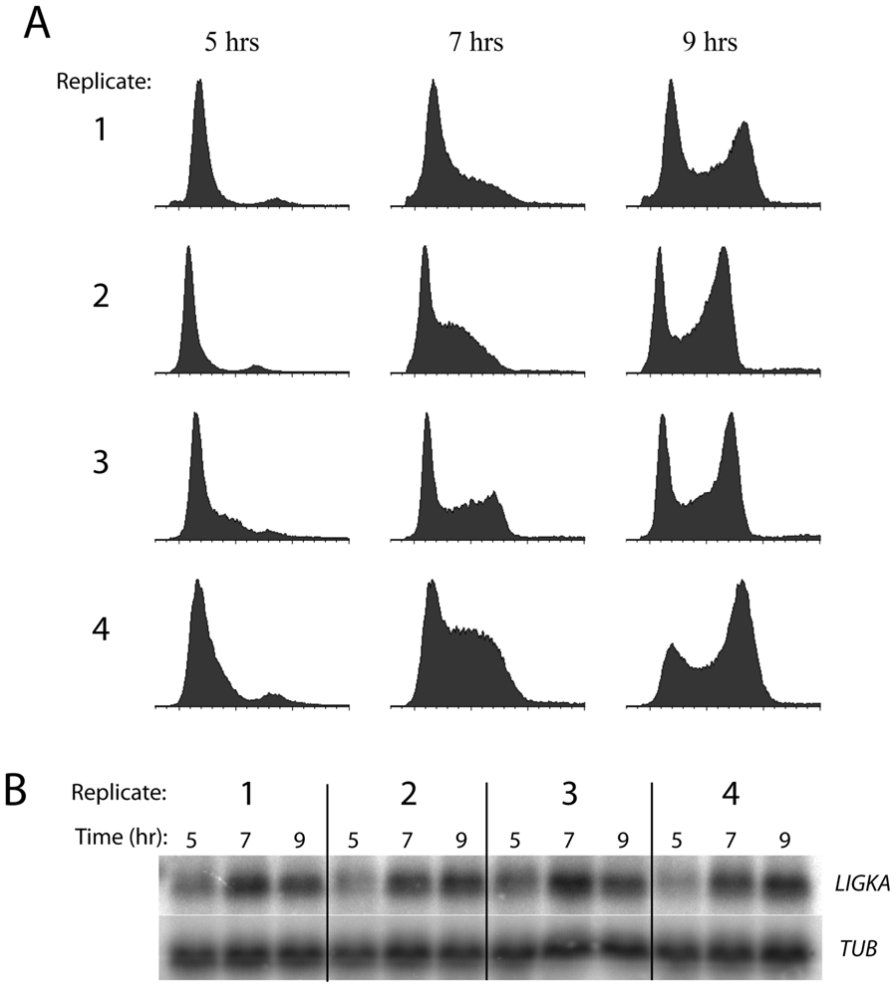
Synchronization of procyclic cells by starvation. Four biological replicates were performed in which cells were released from starvation at time t = 0 and samples collected for flow cytometry and RNA isolation at t = 5, 7 and 9 hours post-release. **A:** Flow cytometry profiles of synchronized procyclic cells. Propidium iodide fluorescence (indicating DNA content) is measured on the *x*-axes and cell count is plotted on the *y*-axes. **B:** Northern hybridization of a known cell-cycle regulated transcript (*LIGKA*) against RNA from synchronized cells.

Procyclic (PC; insect-form) *T. brucei* cells were synchronized by starvation (starve-synch) and induced to resume the cell cycle by dilution into fresh media as described previously [16], [18]. For four independent biological replicates, RNA was isolated at three time points (5, 7 and 9 hours after starvation release). Cells were allowed to recover from starvation for five hours prior to taking the first time-point to avoid finding transcripts that might be differentially regulated primarily due to starvation recovery. As shown by flow cytometry, the cells had indeed recovered from starvation at this time-point as they were just commencing DNA synthesis in most replicates (Fig. 1A). At 7 h, the cells were in late G1/S, and at 9 h, mostly in G2/M. RNA was analysed by Northern blotting and a characterized cell-cycle-regulated transcript, LIGKA [16], [21], was probed for as a positive control (Fig. 1B), and exhibited regulation. RNA was then converted to labeled cDNA, and hybridized to microarrays using the 5-hour time point as a reference sample for the other two time points. After normalization and quality control, the 7 hr/5 hr and 9 hr/5 hr log-ratio values were calculated. The measured magnitude of regulation was rather small for the majority of these genes (<∼2-fold), perhaps due to the incomplete synchronization and variability inherent in the starvation method, or the limited sensitivity of oligo-based microarray detection. For these reasons, and also because earlier time-points were problematic due to the possibility of starvation perturbing gene expression, we investigated alternative strategies for cell synchronization and for subsequent expression profiling with the aim of cross-validating candidate cell-cycle-regulated transcripts.

### Development of a double-cut elutriation procedure for selecting recently-divided cells

Counterflow centrifugal elutriation is a method for accurately isolating cells and other particles by sedimentation rate. This is achieved by subjecting them to two opposing forces: an outward-directed centrifugal force and an inward-directed flow of the suspension fluid. In an elutriation centrifuge, smaller particles are washed out of the elutriation chamber first, and by gradually increasing the fluid flow rate or decreasing the rotational speed, incrementally larger particles emerge. Relatively small size differences can be resolved, such that log-phase cell cultures can be fractionated into populations of cells at particular phases of the cell cycle [22], presumably with the proviso that the cell cycle phase correlates well with cell size for the cell line used. To select cells of a similar age from log-phase cultures of trypanosomes, we developed a non-perturbing procedure, “double-cut” elutriation (DCE), in which the requirement for a uniform relationship between cell size and cell cycle phase is relaxed, and elutriation time is also reduced.

In initial experiments, log-phase PC cell cultures were size-separated by conventional counterflow centrifugal elutriation (sorting of bloodstream-form trypanosomes was rather inefficient under the conditions tested, presumably due to their smaller size or different morphology). Cells were elutriated with a gradually increasing flow-rate, and the first and last fractions emerging from the chamber contained cell populations that were mainly in G1 and G2/M phase respectively (Fig. S1A). However, intermediate flow-rates yielded rather more mixed populations, and obtaining an S phase-enriched population proved elusive. Thus, cell size may only roughly correlate with cell-cycle phase in PC cells, a problem that is perhaps not confined to trypanosomes. Continued culturing of the extreme largest or smallest PC cells resulted in progression through the cell cycle in a reasonably synchronous manner (data not shown), but we required more efficient synchronization to generate a definitive expression profiling data-set.

To improve the separation, we performed a two-step procedure. We first obtained a cell population containing the largest ∼30% of the cells in a log-phase culture by elutriation at a fixed flow-rate (the cells retained at 24 ml/min) and cultured them for one hour. At this point, they were elutriated a second time at a lower flow-rate to select the small cell population (cells not retained at 21 ml/min) that had undergone cell division within the preceding hour (Fig. S1B). This yielded a population of very pure, non-arrested G1-phase cells. Upon further culture, these cells proceeded through the cell cycle with very good synchronicity and with none of the delay seen with starvation, entering S-phase two hours earlier and completing the cell cycle by 9 hours after the second elutriation (Fig. S1C). Beyond this point the cells began to lose synchronization (not shown).

### Expression profiling of DCE-selected cells

**Figure 2:**
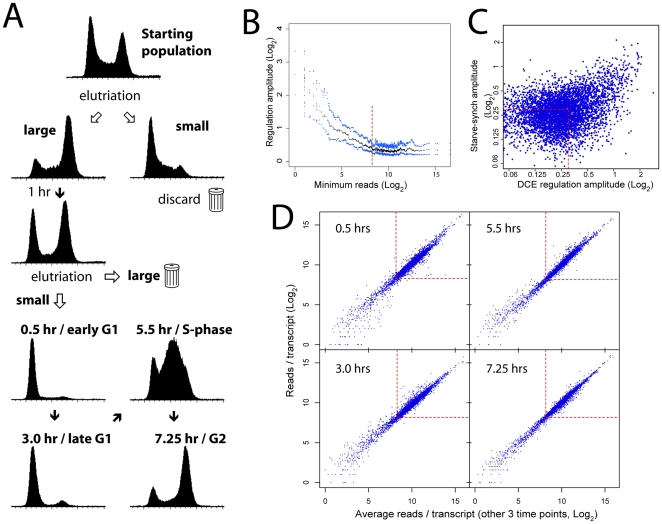
Expression profiling by DCE and RNA-seq. **A:** Flow cytometry profiles of procyclic cells throughout the DCE selection procedure. Propidium iodide fluorescence (indicating DNA content) is measured on the *x*-axes and cell count on the *y*-axes. **B:** Regulation amplitude (the difference between the maximum and minimum expression value throughout the cell cycle) was calculated for each gene. After ranking genes according to minimum read count, the median (black) and upper and lower quartile (blue) amplitude values across a moving window of 100 genes was calculated. Genes with fewer than ∼300 reads in any time point (red line) gave amplitudes that were most likely to be a function of sequencing effort and were therefore excluded from further analysis. **C:** Comparison of gene regulatory amplitude between the starve-synch/microarray-analyzed cells (time points 5, 7 and 9 hrs) and DCE-synch/RNA seq-analyzed cells (time points 3, 5.5 and 7.25 hrs). Genes passing quality control in the RNA-seq experiment and with less than 1.23-fold regulation (0.3 log_2_ units - red lines) in both experiments (red box) were selected as a non-regulated control group for subsequent UTR sequence analyses (motif searching). **D:** Comparison of read counts per transcript for each time-point with the average read counts from the other 3 time points. Red boxes contain transcripts with >300 reads.

We performed a preparative-scale DCE experiment, this time isolating cell populations from four time-points post-selection: early (0.5 hours) and late (3 hours) G1 phase, S-phase (5.5 hours), and G2 phase (7.25 hours), each population consisting almost entirely of the desired cell cycle stage (Fig. 2A). Poly-A+ RNA from these cells was selected and subjected to expression profiling by RNA-seq. Between 13 and 17 million high-quality reads per time-point could be mapped to the *T. brucei* genome.

Transcript boundaries for all genes were assigned to the genome using spliced-leader mapping or *in silico* predictions (see Methods) and the proportions of reads mapping to these transcripts were used to calculate changes in expression. The amplitude of regulation (the difference between the maximum and minimum expression values across the cell cycle) was calculated for all genes and was found to be independent of sequencing coverage for genes with more than ∼300 reads (Fig. 2B). Therefore, transcripts with at least 300 mapped reads in all time-points were retained in the dataset for subsequent analysis (7240 out of 10123 annotated genes). After removal of extraneous copies of multi-copy genes using a previously published list [23], 6949 genes remained (Table S1).

### Comparison of DCE to starvation-synchronization experiments

**Figure 3:**
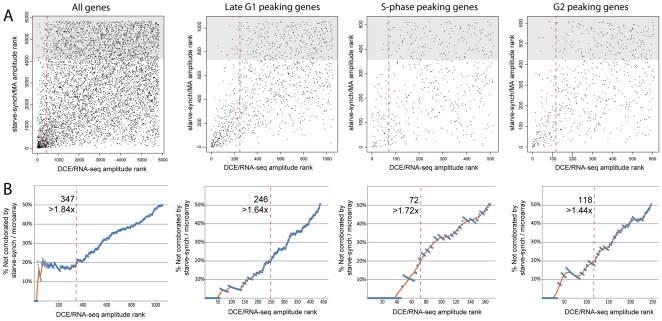
Comparison of genes ranked by regulation amplitudes, between cells synchronized by DCE-selection (RNA-seq) or starvation (microarrays). **A:** Genes were plotted according to their regulation amplitude rank within each dataset, such that the most highly regulated gene was ranked as #1 and so on. Only genes peaking at equivalent times between the two experiments were plotted in the right three panels; all genes were plotted in the left panel. Genes ranking in the least-regulated one-third of the starve-synch dataset (shaded area) were assumed to be representative of genes whose regulation in the DCE experiment was not corroborated by the starve-synch experiment. Red line: 20% non-corroboration threshold derived in B. **B:** A threshold gene-rank in the DCE/RNA-seq dataset (horizontal axis) was varied incrementally and the number of non-corroborated genes inside this cut-off rank was estimated as three times the number of the included genes that were also in the lowest-ranked one-third of the MA data (A; shaded area). For each cut-off rank, the percentage of included non-corroborated genes was estimated (blue circles) with a moving average (orange line). The red dashed line (A and B) indicates the point at which 20% of discovered genes (i.e. to the left of the line) were non-corroborated; the number of genes included using this cutoff rank is indicated. This could be an over-estimation of the false-discovery rate, as genuinely regulated genes might not be detectably regulated in the starve-synch experiment due to differences in the experimental setup.

A comparative plot of regulation amplitudes of the 5844 genes passing quality control in both datasets showed reasonable similarity in regulation amplitude between starve-synchronized and DCE-selected cells, given the differences in synchronization and detection methods and the time-points analyzed (Fig. 2C; R-squared  = 0.25). The most highly regulated genes in the DCE experiment gave regulation amplitudes of about 4-fold, and gene regulation was 1.4× higher than that seen in the starve-synchronized cells analysed by microarray. Gene amplitude rankings were comparable between the datasets: out of the top 347 regulated genes in the DCE/RNA-seq dataset (with >1.84-fold regulation), ∼80% were corroborated by the starve-synch/microarray dataset (Fig. 3, left). The extra time-point included in the DCE experiment (early G1) may account for some high-ranking genes that were not detected as regulated in the starve-synch experiments. Comparison of the times of peak expression values showed a strong correlation between datasets: out of 771 genes with amplitude >1.5-fold that peaked in either late G1, S-phase or G2/M phase in the DCE experiment, 72% peaked at the equivalent time in the starve-synch experiments (Table 1). The genes that peaked at equivalent times in both experiments also showed a better correlation of regulation amplitudes between the two datasets when analysed as separate groups (Fig. 3, right three panels) rather than all together. Thus, we were able to collect some additional corroborated genes from these three subsets of genes. All together, a total of 546 genes were found to be regulated accepting a non-corroboration rate of 20% in the starve-synch/microarray experiments (thus containing ∼435 genuinely regulated genes). Of these, 273 peaked in late G1, 98 in S-phase and 120 in G2 phase in the DCE experiment. At least 55 genes were found to peak in early G1; however, this is likely to be an underestimate as we could not directly cross-validate this time-point with a specific corresponding time-point from the starve-synch data. While the microarray results were useful to cross-validate the DCE/RNA-seq data, RNA-seq allowed analysis of about 1000 extra transcripts with expression levels too low to give reliable results on the available microarrays (Table S1; these genes were excluded from the corroboration tests above). Therefore, the expression values from the DCE/RNA-seq dataset were used for all the following analysis.

**Table 1:** Comparison of gene expression peak times for two different synchronization techniques.

starve- synchDCE-selected	Late G1	S-phase	G2/M	total genes
**Early-G1**	50	43	**106** (1.7x)	199
**Late-G1**	**323** (1.8x)	54	35	412
**S-phase**	27	**137** (2.3x)	67	231
**G2/M**	16	19	**93** (2.3x)	128
**total genes**	416	253	301	970

Contingency table showing the number of genes peaking at each of the three time-points in the starve-synch/microarray experiments and the four time-points from the DCE/RNA-seq experiment for genes with >1.5-fold regulation amplitude. Shown in brackets is the fold-difference relative to the expected number of genes that should be present in each microarray category by chance.

### Gene function and cell cycle regulation

The extent of overlap between our data and data from other organisms was determined. Consensus lists of cell-cycle regulated transcripts were obtained from Cyclebase [24], using studies in *S. cerevisiae* [25], [26], [27], [28] and human cells [29]. The estimated number of cell-cycle regulated genes in human and yeast (∼600 each [24]) were broadly similar to our estimate of about 546 for *T. brucei.* However, only ∼15% or 25% of the regulated *T. brucei* genes that had a homologue in human or yeast, respectively, were also cell-cycle regulated in those organisms (Table 2). This was only double the extent of overlap expected by chance (*p*<10^−4^ for both organisms; χ^2^ test). The majority of regulated genes in *T. brucei* could not have been predicted from homology to regulated genes in humans or yeast. This presumably reflects the large evolutionary distance, and differences in the cell biology between kinetoplastids and opisthokonts. Notably, unlike kinetoplastid organisms, opisthokonts in general do not coordinate the replication of their organelles with the cell cycle.

**Table 2:** Comparison of cell-cycle regulated genes between *T. brucei* and other eukaryotes.

	*T. brucei* regulated	*T. brucei* non-reg	sum
**Human regulated**	20 (6)	92 (106)	112
**Human non-reg**	110 (124)	2136 (2122)	2246
**Human, sum**	130	2228	2358
***S. cerevisiae*** ** regulated**	39 (19)	291 (311)	330
***S. cerevisiae*** ** non-reg**	120 (140)	2376 (2356)	2496
***S. cerevisiae*** **, sum**	159	2667	2826

Putative orthologues of *T. brucei* genes in yeast and human were identified by one-way BLAST searches (score >50). Cell cycle regulated or non-regulated *T. brucei* transcripts were further classified according to whether their orthologues in each organism were marked as being periodically expressed in Cyclebase [24] and the numbers of genes in each category are given. In brackets is the number expected in each category by chance.

**Figure 4:**
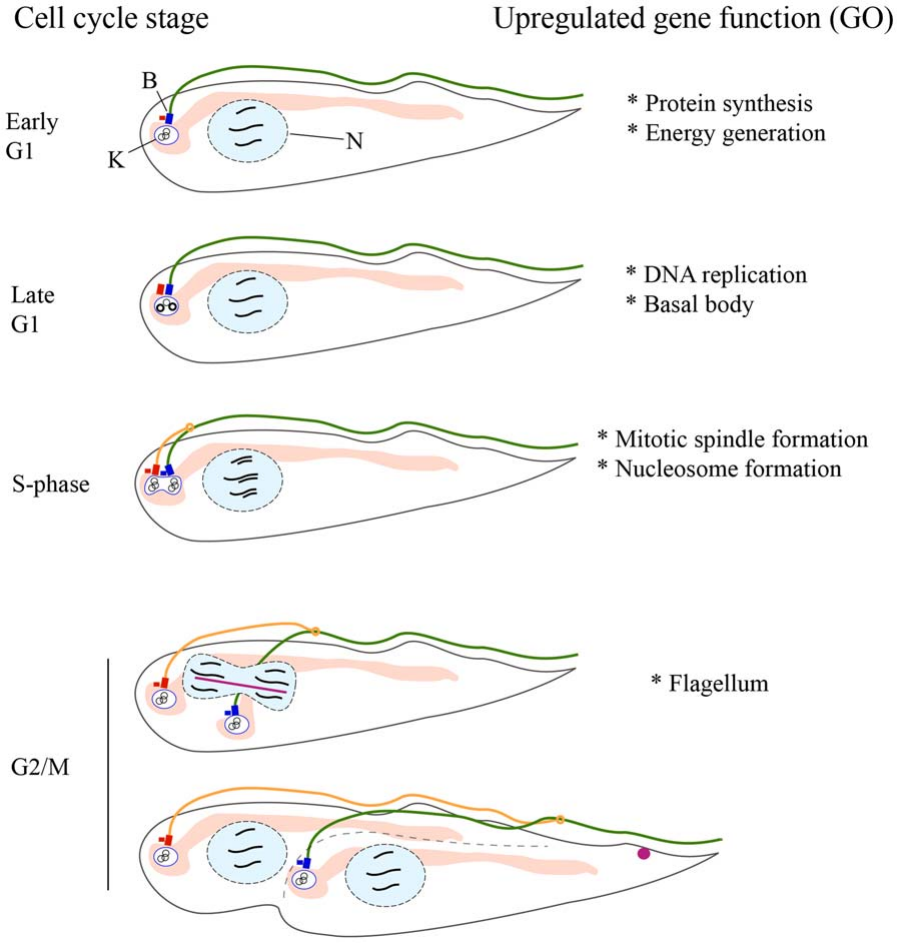
Relationship between formation of cellular components and cell-cycle regulated gene expression. Right: summary of functions of upregulated genes as suggested by GO analysis. Left: Representation of procyclic *T. brucei* cells progressing through the cell cycle. K: the kinetoplast (organelle containing the mtDNA, which consists of many circular DNA molecules). N: Nucleus. B: Basal body (blue rectangle) and pro-basal body (red rectangle). In light pink is the mitochondrion; in green is the old flagellum, which emerges from the posterior end of the cell (left) but is tethered to the dorsal side of the cell along its length. In late G1 phase the probasal body (small red rectangle) matures into a new basal body which will localize the base of the new flagellum, and the kinetoplast is already in S-phase. In S-phase, the new flagellum (orange) begins to elongate, anchored to the old flagellum by a mobile flagellar connector structure (orange circle) [34], while the kinetoplasts and basal bodies separate. After DNA replication an intranuclear mitotic spindle forms (pink line) and cells enter mitosis. Before cytokinesis, the Chromosomal Passenger Complex relocates from the spindle to the cell anterior (pink circle, bottom panel) where it moves with the cytokinetic furrow from anterior to posterior (dotted line) [48].

To find common biological themes amongst cell-cycle regulated *T. brucei* genes, the 546 putative regulated genes were interrogated for over-represented Gene Ontologies (Table S2; summary in Fig. 4). In early G1 phase, transcripts encoding enzymes for energy generation and components of the mRNA translation apparatus were somewhat upregulated; perhaps related to the need for general cellular growth in this phase. mRNAs peaking in late G1 encode a large number of proteins involved in DNA replication, as well as two proteins of the basal body, which matures from a pro-basal body at the G1/S-phase transition. During S-phase, histones and microtubule-based motor proteins were upregulated, probably reflecting the imminent need for DNA packaging into chromatin and the assembly of a mitotic spindle, respectively. Lastly, in G2/M, flagellar proteins were upregulated.

**Figure 5:**
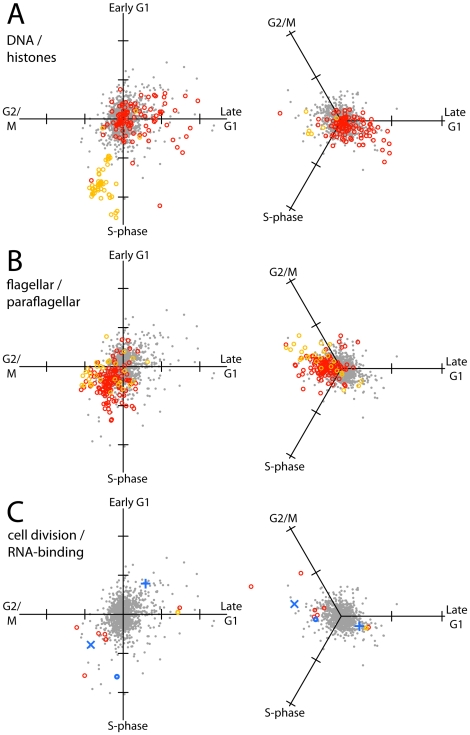
Vectorial representation of regulation of selected functional groups of transcripts in the cell cycle, from DCE/RNA-seq data (left panels) or starve-synch/microarray data (right panels). Each time-point in the cell cycle that was analyzed was arranged as a vector pointing outwards from the origin. Gene expression values were plotted by vector addition; tick marks on axes are one log_2_ unit. Reference profiles from 1000 randomly selected genes are plotted in grey; genes in specific functional groups are plotted as coloured circles. **A:** Red: Transcripts annotated with “DNA” in “product description” or in gene ontology fields of the TriTryp database (relevance score >40). Orange: Histone-encoding transcripts. **B:** Red: Transcripts encoding flagellar proteins [32]. Orange: Transcripts encoding Snl2-dependent paraflagellar rod proteins [33]. **C:** Putative mediators of mitosis and cytokinesis, and RBPs. Red: Transcripts encoding Aurora kinases and chromosomal passenger complex proteins. Orange: Polo-like kinase. Blue: Selected RBPs. (×) PUF9, (+) RBP45 homologue, (o) DRDB17.

The existing GO databases lack a significant amount of gene information that is available from experimental studies. We therefore investigated in more depth the temporal regulation of genes involved in the assembly of two major cellular structures that are assembled at specific points in the cell cycle: chromatin and the flagellum. To represent the expression profiles of these gene groups graphically, the three (starve-synch) or four (DCE) expression values for each gene were added together as orthogonal vectors on a 2D plot to appraise the approximate timing of peak expression (Fig. 5; note that the angle from the vertical does not linearly correlate with the timing of the expression peak in the cell cycle, especially for the starve-synch dataset in which all time-points are somewhat closer together).

To classify genes involved in DNA replication, text searches for “DNA” were done on *T. brucei* protein-coding genes in the GeneDB database (see Methods; Table S3). These genes encode DNA polymerases, topoisomerases and DNA ligases, and include many transcripts that had been previously suggested to be under the control of CSBPs [30]. Out of the 18 transcripts in this group regulated at greater than 1.9-fold, 15 peaked at around late G1 phase or early S-phase, when rapid production of proteins involved in DNA replication are needed; the other 3 peaked in late S-phase (Fig. 5A, red circles). Histones, which are only required after replication is complete, peaked later, at around the end of S-phase (Fig. 5A, orange circles). Although the mitochondrial genome is replicated slightly earlier in the cell cycle [31], we could not discern any peak time difference between mtDNA replication proteins and nuclear DNA replication proteins at the temporal resolution used in these profiling experiments (not shown).

To classify genes encoding flagellar proteins, experimental data from two proteomic studies were employed, grouping genes encoding flagellar proteins into two classes: “general flagellar proteins” [32], and “Snl2-dependent paraflagellar rod proteins” [33] (see Methods and Table S3). These transcripts exhibited higher amplitudes over the cell cycle, compared to all other genes (*p*<10^−15^, Mann-Whitney U test), and as a group their regulation was strikingly uniform and pervasive (Fig. 5B). Rather than peaking sharply in G2/M, flagellar protein mRNAs exhibited a broad peak over S- and G2/M phases, which correlates with the period of flagellar elongation that commences upon segregation of the kinetoplast (mtDNA) during nuclear S-phase and continues to G2/M phase [34]. The maturation of the flagellum is important in the later stages of the cell cycle of bloodstream-form trypanosomes, in which flagellar beating is required for cytokinesis [35].

We next investigated other proteins having regulatory functions, especially conducting a literature search for protein complexes that control mitotic spindle formation and cytokinesis in *T. brucei*. We found that all three Aurora B kinase homologues were highly regulated at the transcript level (1.8, 2.3 and 3-fold, respectively), as were other proteins found in the chromosomal passenger complex, in which TbAUK1 is found (Fig. 5C, red circles) [36]. Another player in mitotic spindle formation and cytokinesis, Polo-like kinase, was also observed to be regulated but peaked earlier (Fig. 5C, orange circle), which is consistent with its temporal expression pattern at the protein level [37], while the Tousled-like kinases were not regulated at all (see Discussion). Because RNA-binding proteins (RBPs) play a central role in transcript-level regulation, we also investigated whether they were themselves regulated at the transcript level and found at least three where this could be the case (Fig. 5C, blue symbols): PUF9, CSBPII component Tb927.5.760 (both of which themselves mediate cell-cycle coupled mRNA regulation) and uncharacterized RRM-containing protein Tb927.8.710 (DRBD17).

### Searching for *cis*-regulatory motifs

Due to polycistronic transcription in *T. brucei*, transcriptional regulation, if present, should cause co-regulation of adjacent transcripts. There was no evidence for this: of the 546 putative regulated transcripts, only ∼10% were immediately upstream of another regulated gene. Further, we calculated a moving average of regulation along all chromosomes and compared this to 1000 randomly shuffled genomes, but found only one significant cluster of 11 regulated genes located close together on chromosome 1 (see Fig. S2 and Discussion). Even for this group of regulated genes, transcriptional regulation seems doubtful because expression peaked at different times in the cell cycle for the different genes, and many flanking genes in the same polycistronic gene array were not regulated. Also, a cross-comparison with the first or last genes in polycistronic units [38] revealed no significant intra-polycistron location bias for cell-cycle regulation (not shown). Therefore we turned to post-transcriptional regulation as the most likely regulatory mechanism. Post-transcriptional regulation requires a signal on the target mRNA, usually in the form of sequence or structural motifs present in the UTRs that are binding sites for a *trans*-acting factor. To find possible *cis*-regulatory elements, we first estimated the 5′ and 3′ UTR sequences for each gene (see Methods) and then analyzed co-regulated clusters of genes for over-representation of any sequence motifs.

**Figure 6:**
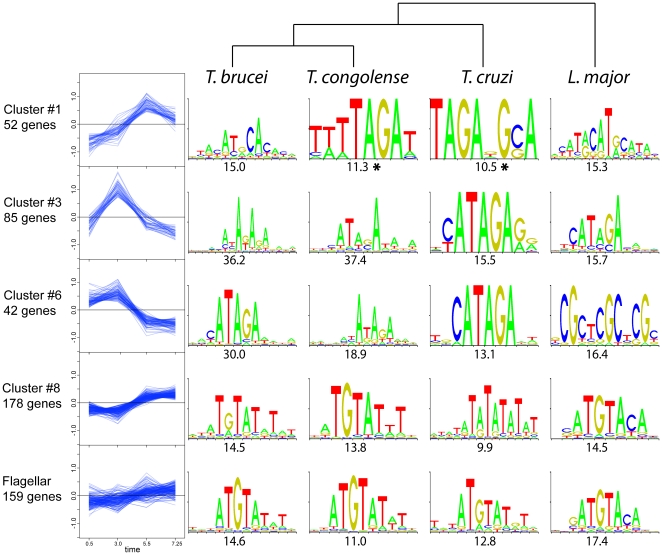
Identification of over-represented sequence motifs among selected clusters of co-regulated transcripts. Cluster expression profiles from DCE/RNA-seq data are plotted on the left, with the log_2_ expression values on the *y* axis and time on the *x* axis. The top-ranking over-represented sequence motifs from UTR sequences in four kinetoplastid species, as found using Trawler, are presented on the right with the *z*-score below. (*) indicates motifs supported by MEME analysis of Cluster #1 (see Table S5). Top: a dendrogram showing the phylogenetic relationship between species.

From the DCE experiment dataset, genes were grouped according to temporal expression profiles by K-means clustering using the TM4 software [39] and an expected cluster number of 20. Out of these 20 clusters of genes, 7 that showed significant regulation were selected for sequence analysis (Table S4). Non-regulated genes (amplitude<0.3 log_2_ units in both starve-synch and DCE-synch experiments – Fig. 2C) were used as a control set of sequences. The Trawler software [40] was then used to identify over-represented motifs in each cluster, relative to the control set. The evolutionary conservation of these motifs was also analyzed by first assembling presumptive UTR sequences of annotated genes in *T. congolense*, *T. cruzi*, and *Leishmania major* using Splicemodel predictions, and then assembling the sequences into regulated groups based on the assumption that these orthologous genes are regulated in the same way as in *T. brucei*. Despite the likely sources of noise inherent in estimating the UTR sequences and the absence of orthologues of some genes, the same motifs were identified *de novo* from the predicted regulated sequences in the four kinetoplastid species examined (Fig. 6).

The top-ranked motifs from four clusters that produced conserved motifs are shown in Fig. 6. Cluster #1, with a late expression peak in S-phase, gave a short, novel [UAGAU] motif, but it was only the top-ranked motif for two of the four species (Fig. 6, top row). To better clarify the significance of this motif, we re-analysed cluster #1 using MEME, an alternative algorithm that has a ‘zero or one occurrence per sequence’ mode which reduces bias towards motifs that are repeated many times in only a small fraction of the sample sequences. We also assembled cluster #1 sequences from a further four kinetoplastid species, and found that all eight kinetoplastid species analysed with MEME gave a variant of [UAGAU] as the best-scoring motif for this cluster (Table S5). For clusters #3 and #6 (which had similar expression profiles), analysis with Trawler yielded motifs that were variants of the well characterized canonical Cycling Sequence (CS) with consensus [CAUAGAAG] [12]. Transcript cluster #8, despite showing relatively subtle regulation, contained a well-conserved [AUGUAU*U] motif. This cluster also contained a large number of transcripts encoding flagellar proteins, and reiterating the motif search using the flagellar proteome recovered the same motif (bottom row, Fig. 6).

In general, there was no apparent bias towards the 5′ or 3′ UTR for most of these motifs in *T. brucei*, consistent with the previous observation in *C. fasciculata* that the canonical CS is functional in either location [41] and sometimes even in the pre-mRNA [42]. The exception to this was the cluster #8/flagellar motif [AUGUAU*U], which, on a per-nucleotide basis, occurred ∼2.5×more frequently in the 3′ UTRs than in the 5′ UTRs (81 and 1681 occurrences on the 5′ and 3′ UTRs respectively; while average predicted UTR lengths in cluster #8 were 182 and 1412 nt). The conservation of sequence motifs in these regulated transcripts amongst kinetoplastids reinforces their likely biological significance.

## Discussion

### Utility of Double Cut Elutriation

We considered various alternative existing methods for cell synchronization apart from starvation. Although drugs such as hydroxyurea can be used to effectively synchronize cells at the level of DNA synthesis [20], [43], [44], the mechanism of action (deprivation of nucleotide precursors via DHFR-TS inhibition) is somewhat similar to the general nutrient deprivation technique we had already employed, and might similarly cause changes in starvation- or stress- regulated transcripts. There are also concerns with drug-mediated synchronization about de-coupling of DNA synthesis (“nuclear” cell cycle progression) from cyclin activation (“cytosolic” cell-cycle progression) [45] as well as potential toxicity. Flow cytometry using cell-permeant DNA dyes can be used to sort cells according to DNA content [46], [47] but cannot separate early- from late- G1 stage cells and the yield is limited. Elutriation, in contrast, can yield preparative amounts of synchronous cells, limited mainly by the size of the elutriation chamber used. Although conventionally, fractionation is done by gradually increasing flow rates and isolating populations of incrementally larger cells, we found that by selecting for large, then small cells over a given time-frame, a very pure age-specific population of cells can be obtained that immediately begin to progress through the cell cycle. The trade-off between yield and selection stringency could be adjusted by altering the time between the first and second elutriations. Another advantage is the bias towards actively dividing cells, because quiescent cells, if present, would be removed in one of the two elutriation steps.

### Differential expression of genes important in mitosis, cytokinesis and RNA stability

The GO terms associated with genes that were upregulated at certain times during the cell cycle generally correlated with the biological processes occurring a short time later (summarized in Fig. 4). The transcriptome at each point in the cell cycle gives an overall picture of the processes that occur at specific points. Many of these are unique to the kinetoplastids, for example the replication of the mtDNA just before nuclear S-phase and the flagellar growth occurring in G2 phase. Others are common to all eukaryotes, such as DNA replication. A large number of genes with no known orthologues in other model eukaryotes exist in the kinetoplasts and these are largely uncharacterized. That some of these orphan genes are regulated over the cell cycle gives a clue that they may function in cell proliferation [30], which could expedite the search for suitable drug targets to combat the diseases they cause.

Surprisingly, when we initially investigated whether cell cycle regulation proteins such as cyclins and cyclin-dependent kinases in *T. brucei* might be regulated at the transcript level as in yeast [1], we found that while one or two were regulated, there was no significant overall enrichment for these genes amongst the regulated transcripts (not shown). However, some proteins that spatially regulate mitosis and cytokinesis did show regulation as a group (Fig. 2C). Aurora kinase 1 (TbAUK1) forms a complex with four other chromosomal passenger complex (CPC) proteins that facilitates spindle formation during mitosis and then rapidly re-locates to the cleavage ingression during cytokinesis to guide cell scission [48] (Fig. 4). The co-expression of both TbAUK1 and TbAUK2 and the other four known CPC proteins, peaked at around late S-phase and G2 phase, coinciding with their biological functions. However, expression of the third Aurora kinase orthologue (TbAUK3/Tb09.160.0570), which has not been characterized in depth, peaked much earlier (before S-phase), suggesting a role for this kinase independent of the CPC. Polo-like kinase (Tb927.7.6310) also peaked early (i.e. before S-phase) for a G2/M phase regulating protein, but this might be explained by additional functions for this kinase earlier in the cell cycle, such as basal body maturation and kinetoplast segregation [49]. On the other hand, the two *T. brucei* tousled-like kinases that are targets of TbAUK1 and facilitate S-phase progression and spindle formation [50], were not regulated at the transcript level. Thus, mRNA-level regulation seems to control expression of some, but not all, effectors of mitosis and cytokinesis.

The mechanism of post-transcriptional regulation of these transcripts almost certainly involves differential activity of sequence-specific RNA-binding proteins (RBPs) over the cell cycle. However, predicted RBPs did not feature prominently on the list of regulated transcripts, which argues against a general model of cell cycle-coupled regulatory cascades in which RBPs bind to each other's transcripts and regulate transcript stability (although regulation of translation is one possibility that we did not investigate). Perhaps post-translational modifications such as phosphorylation regulate RBP activity, as has been suggested for the CSBP II complex [15]. Two notable exceptions to this lack of transcript-level regulation of RBPs are PUF9, an RBP that mediates cell-cycle regulation of a small group of downstream targets [16], and which was observed here to be regulated at the transcript level by 1.9-fold; and Tb927.5.760, the homologue of the *C. fasciculata* RBP45 subunit of CSBPII which is also responsible for cell cycle regulation of cognate transcripts [15], showing upregulation of ∼1.9 fold in early G1 phase. While insufficient to explain the stronger regulation displayed by some of the target mRNAs of these RBPs, these mRNA-level changes could synergize with other modes of cell-cycle coupled regulation of these RBPs (e.g. phosphorylation, degradation) to maintain tighter coordination between cell cycle phase and the expression of their downstream target transcripts. The RNA encoding a third putative RBP, the uncharacterized RRM-domain protein Tb927.8.710 (DRBD17), was 3-fold regulated, peaking in S-phase; this protein has not been implicated in cell-cycle regulation of any RNAs to date.

### Candidate *cis*-regulatory UTR motifs

Transcriptional regulation, if present, would be expected to result in co-regulation amongst co-transcribed genes. While a small number of cell cycle-regulated genes were clustered together in a section of chromosome 1 (Fig. S2), not all genes on this polycistronic transcriptional unit were regulated, ruling out transcriptional control as a general mechanism for regulation.

Over-represented sequence motifs among co-regulated genes are good candidates for binding sites of trans-acting regulatory factors that act as post-transcriptional regulators. The [CAUAGA] motif of late G1-upregulated clusters #3 and #6 (Fig. 6), homologous to the well-characterized canonical Cycling Sequence (CS) of kinetoplastids [12], is known to be recognized by the CSBP II complex that mediates regulation of a cohort of transcripts that mostly encode proteins involved in DNA metabolism. Here, the presence of this motif was found to be correlated to cell-cycle regulation of late G1-phase peaking transcripts. However, there was a fairly high degree of variation in terms of the timing of regulation amongst transcripts containing this motif, suggesting that other motifs or structural features modulate regulation. It is perhaps noteworthy that the CS Binding Proteins that were originally found to bind this sequence, CSBP A and B [13], were later found to be dispensable for regulation of the particular transcripts analysed [14]. One possibility is that a modulatory role for these proteins might exist for some but not all of these regulated transcripts, depending on structural context or other cofactors.

A short [UAGAU] motif was found to be over-represented in co-regulated cluster #1, which contained highly regulated genes that peaked later in the cell cycle. While this short motif is expected to occur quite often by chance, it occurred significantly more frequently in putative S-phase-specific transcripts in eight kinetoplastid species (Table S5). The similarity between this motif and the 3′ end of the canonical CS [CAUAGA] may be significant, despite the ∼2.5 hour delay in peak expression for cluster #1 relative to the late G1-peaking transcripts. It is possible that the binding specificities of some of the CSBPs are altered over the cell cycle, or that weaker binding of this suboptimal CS is mechanistically important, e.g. allowing binding competition or synergy to occur between multiple CSBPs, or binding of a divergent CSBP. Alternatively, the motif may be targeted by an entirely different set of factors. Further investigation is required to determine the importance of this motif for regulation of these transcripts, and to identify the relevant *trans*-acting factors.

### A post-transcriptional regulon of transcripts encoding flagellar proteins

The [AUGUAU*U] motif found in co-regulated cluster #8 contains a likely PUF-family RNA-binding domain cognate core, [UGUA] [51]. The transcript clusters containing this motif appeared to have a broad peak in expression across S-phase and G2-phase. Although regulation was relatively modest, the motif was quite well-conserved across kinetoplastid evolution. Cluster #8 encodes a large number of flagellar proteins, consistent with the timing of rapid flagellar elongation during the S and G2 phases [34]. Remarkably, when transcripts were clustered according to flagellar proteomics data instead of cell-cycle co-regulation, and the motif search was reiterated, the same [AUGUAU*U] motif was consistently obtained from all kinetoplastid species (Fig. 6, bottom row). Individual PUF proteins have been shown to bind to mRNAs targeted to particular organelles in yeast [52], so the presence of a PUF recognition-like motif in these transcripts could be a correlate of general co-regulation of flagellar protein synthesis, including but not limited to cell-cycle regulation. Regulation of flagellar proteins is common in kinetoplastids. We have already shown, using microarrays, that ∼20 mRNAs encoding *T. brucei* flagellar proteins are diminished during differentiation-associated growth arrest [7]. The *T. brucei* flagellum undergoes dramatic elongation during differentiation of the highly motile migratory epimastigote that travels from the foregut to the salivary gland of the tsetse fly, and is also used for attachment to the salivary gland epithelium in the metacyclic forms [4]. In the intracellular forms of Leishmania spp. and *T. cruzi*, the flagellum is even more dramatically regulated, being reduced to a short stub without motile function. Interestingly, an element within the 10 nt sequence [AUGUAUAGUU], which contains a remarkably similar 5′ sequence to the motif from cluster #8 in *T. brucei*, was found in the UTRs of paraflagellar protein mRNAs in *Leishmania mexicana* [53], [54]. This sequence element promotes mRNA destabilization in the non-flagellated mammalian (amastigote) stage. Thus, the [AUGUAU*U] motif may be related to sequence elements involved in differentiation-associated mRNA regulation in Leishmania, possibly even binding to orthologous trans-acting factors. The combination of co-regulated expression, functional relatedness and common *cis*-regulatory motifs satisfies all the criteria for kinetoplastid flagellar proteins to be considered as a post-transcriptional regulon.

### Concluding remarks

Post-transcriptional regulation is of prime importance in both differentiation and proliferation of kinetoplastid parasites. While the interactions between RBPs and key signature sequences in mRNA transcripts are likely to be an essential for regulation, a large number of regulated transcripts lack any putative *cis*-regulatory sequence motif. Very small co-regulated groups (such as the four identified PUF9-associated transcripts [16]) were missed in this analysis due to the relatively large numbers of transcripts assigned to each cluster. Biochemical evidence of interactions with RBPs and other experimental validation will be required to confirm co-regulation of these transcripts. More advanced algorithms are also needed to search for motifs consisting of sequence/structure combinations. Nonetheless, the putative *cis*-regulatory elements identified here are good starting points for investigating the mechanisms driving periodic gene expression over the kinetoplastid cell cycle.

## Methods

### Synchronization of procyclic cells by starvation and microarray analysis

Cell starvation was performed essentially as described [18]. Briefly, cells were seeded at 10^6^ per ml in MEM-pros media, grown for ∼64 hours with gentle rocking and then diluted 1∶5 in fresh MEM-pros medium, auto-conditioned as described previously [55]. For each time-point analyzed after starvation release, an aliquot of approximately 5×10^5^ cells was fixed in 70% ethanol/30% TBS, incubated with propidium iodide and RNase A for 30 minutes at 37°C and analyzed by FACSCAN. RNA was isolated by the Trizol method and analysed by Northern blot or microarray. cDNA labelling and microarray hybridization and was done exactly as previously described [7], using oligo-based microarrays (TIGR). A dye-swap (Cy3/Cy5) was performed for each hybridization. Images of microarray scans were quantified using GenePix. Mean local background pixel intensities were subtracted from mean spot-pixel intensities for both channels, which were then combined as Log_2_-ratios. These values were Loess-normalized (in R) with respect to overall spot intensity within each of the 48 printing-tip blocks on the array. Outlying replicates for each spot were identified as those replicates for which exclusion from the analysis reduced the standard deviation of the log_2_-ratio values by at least 0.2 units: a maximum of one outlying replicate was excluded per gene. Raw and normalized microarray data was deposited as a MIAME compliant entry into the ArrayExpress database (accession #E-MTAB-515). Values from replicates were averaged and genes with a weak signal (median intensity of<8 log_2_ units) were filtered from the dataset.

### Selection of recently divided cells by double-cut elutriation

Elutriation buffer consisted of a solution of HEPES-buffered saline and MEM-pros medium mixed in a 4∶1 ratio, with 1 mM pyruvate. In pilot experiments, 10^9^ procyclic (PC) cells were collected from a log-phase culture by centrifugation, resuspended in 10 ml of elutriation buffer, disaggregated by passing twice through a 20-gauge needle, and injected into the loading chamber of an Avanti J-26 XP elutriation centrifuge equipped with a JE-5.0 rotor (Beckman Coulter). Cells were loaded at<12 ml/min into the elutriation chamber (5 ml capacity) against a constant centrifugal force (4,700×g) at 27°C. In these pilot experiments, the flow rate was then incrementally increased by 2 ml/min for every 50 ml fraction collected, and most cells were seen to emerge from the chamber at flow-rates of between 15 and 32 ml/min (Fig. S1A). Bloodstream-form cells did not separate as efficiently (possibly requiring a higher centrifugal force due to their smaller size), therefore only PC cells were used.

For selection of recently-divided cells by double-cut elutriation (DCE), smaller cells were discarded from log-phase cultures (∼3×10^9^ cells) by elutriation at 4,700×g against a counter-flow of 24 ml/min at 27°C. Retained large cells (∼30%) were then flushed out of the chamber at 35 ml/min and FCS was immediately added to 20% (v/v). These cells were collected by centrifugation, gently resuspended in complete pre-warmed MEM-pros media and cultured at 27°C. After ∼45 minutes, cells were collected and resuspended in elutriation buffer as before, and one hour after the first elutriation, small cells were selected from this population by passing through the elutriator at 4,700×g against a counter-flow of 21 ml/min. These recently (<1 hr) divided cells (∼1.5×10^8^ cells or 5% of the original culture) were cultured and time points taken for the next 11 hours (Fig. S1B, S1C).

### RNA-seq

Poly-A+ RNA was selected by oligo-T chromatography and the RNA was fragmented and size-selected for ∼300 nt fragments. These were reverse-transcribed and a library generated for Solexa sequencing with reads of 72 nt. Raw reads were subject to quality-based trimming of base calls with Phred quality <26 and reads were removed if more than three low-quality base calls were present in the 28 nt 5′ seed region. Remaining reads were mapped to the *T. brucei* genome using Bowtie [56] and aggregated into transcript regions using the Genominator package [57]. Reads mapping to more than one genomic sequence were randomly assigned to one of these locations. Only uniquely mapping reads were assigned to transcripts unless these reads numbered fewer than 400, in which case multiple mapping reads were also counted for that transcript. Values were normalized for the slightly different numbers of total mapped reads from each time point, log_2_-transformed, then normalized to set the average value across all time-points to zero.

### Analysis of gene function

To compare the *T. brucei* datasets to human and yeast, lists of orthologue pairs were generated by searching human and yeast protein databases with each predicted *T. brucei* protein sequence using BLASTx. Using a score cutoff of 50 and expect value of 0.0001, 2358 predicted *T. brucei* proteins were paired to a human gene (2826 for *S. cerevisiae*).

Searching for over-represented terms in the automatically annotated GeneDB Gene Ontology fields was performed using the GOstat program [58], with a *p*-value cutoff of 0.1 and correction for multiple testing (False Discovery Rate, Benjamini). To extract a list of genes functioning in DNA-related processes, genes containing the word “DNA” in the “Product description” and “Gene ontology” fields (but excluding “DNA-dependent RNA polymerases”) were scored for relevance using the TriTrypDB scoring algorithm. Genes scoring above 40, the chosen cut-off value, usually either contained “DNA” in the product description or at least three times among the Gene Ontology entries, and included many DNA polymerase subunits, topoisomerases, and DNA repair enzymes, but not histones. To generate a list of flagellar proteins, we used the mass-spectrometry results of [32], filtering out the proteins that they found to be more highly associated with the flagellar base complex rather than the flagellum itself (abundance ratio >0) to give 153 proteins. 31 paraflagellar rod proteins (some already in the first dataset) were added that had been identified in a comparative proteomics study of *T. brucei* cells lacking a paraflagellar rod [33]. To find proteins with a putative RNA-binding domain, we used the list from [59]. Proteins involved in mitotic spindle formation and cytokinesis were identified through a survey of the literature.

### UTR sequence analysis

All sequences were downloaded from the TriTryp database, version 2.3 except for the *T. brucei* genome, for which version 1.3 was used. To identify likely orthologues, each annotated CDS sequence in the *T. brucei* genome was compared to all currently listed CDS sequences in the *Trypanosoma congolense*, *Trypanosoma cruzi* (except for non-Esmeraldo sequences for which an Esmeraldo-like CDS was already annotated) and *Leishmania major* genomes using one-to-one tBLASTx searches (best hits with score >170). Multiple-copy genes in the *T. brucei* genome were removed using the list from [23].

Transcript extremities were estimated using the observation that individual mRNA sequences on the pre-mRNA (a long polycistronic transcript from which all mature kinetoplastid mRNAs derive) are demarcated by poly-pyrimidine tracts that are recognised by the splicing complex. This complex promotes cutting and polyadenylation of the RNA roughly 70 nt upstream, and also splicing of the invariant 35 nt splice-leader RNA onto the first possible downstream splice acceptor site (AG dinucleotide) [60]. These two events define the upstream gene's 3′ UTR and the downstream gene's 5′ UTR, respectively. To map the 5′ UTRs, uniquely-mapping reads containing part of the invariant 5′ spliced-leader sequence were mapped onto the genome and the median trans-splicing location was selected. We used Splicemodel [60] and in-house Perl scripts to predict the 3′ processing sites for all transcripts (and also those 5′ processing sites for which no evidence was found in our deep-sequencing data, and for other kinetoplast species) by searching for intergenic polypyrimidine tracts. Splicemodel predictions with these parameters was rather inclusive, i.e. it generally predicted 3′ UTRs of lengths equal to, or longer than, the actual length. For genes where mapped polyadenylation sites were available from RNA-seq data [23], ∼41% of predicted 3′ UTR lengths fell within a 15% or 30 nt margin of the real length, while only 14% were shorter.

### Expression Profile Clustering and Phylogenetic Footprinting

The estimated 5′ and 3′ UTR coordinates were used to extract all UTRs from genomic assembly sequences. (Genome sequences were pre-processed to mask tandem repeats using Tandem Repeat Finder 4.0 [61] with input parameters:- match score 2; mismatch penalty 7; indel penalty 8; match probability 80; indel probability 10; min score 45; max period 200.) K-means clustering (set to 20 clusters) implemented in the TM4 software [62] was used to cluster genes into co-expressed groups based on the DCE/RNA-seq expression data. The predicted UTR sequences from these clusters were then used as input for Trawler [63] to identify potential regulatory motifs *de novo*. Unregulated (control) transcripts were assigned as those passing minimum expression thresholds and having expression changes of less than 0.3 Log_2_ units (1.23-fold) in both starvation- and DCE- synchronized cells. Homologous groups of genes (tBLASTx scores >170) from the other trypanosomatids analysed were also collected into clusters, their UTR sequences estimated using Splicemodel and masked using Tandem Repeat Finder [61], and finally used as input for motif analysis with Trawler. For the additional analysis of Cluster # 1 transcript with MEME [64], UTR sequences were further masked for short (> 5 nt) di- and tri- nucleotide repeats using in-house Perl scripts to mask internal repeats. MEME, in the ‘zoops’ mode (zero or one occurrence per sequence), was used to process the sequences to find the best motif between 5 and 8 nt long occurring in at least half of the sequences.

## Supporting Information

Figure S1 **Development of a DCE procedure for isolating synchronous PC cells.** **A:** Cell yield after fractionation of a log-phase PC culture using a constant centrifugal force (4,700×g) and increasing flow-rates. Cells from each fraction were saved for flow cytometry; results for selected fractions are shown. **B:** Schematic for the DCE procedure showing flow cytometry data for each step, from a pilot experiment. **C:** Flow cytometry results taken at various times after commencement of culturing of DCE-selected cells.
(TIF)

Figure S2 **Averaging cell-cycle regulatory amplitude across genomic regions reveals only one small cluster of regulated genes.** All but one copy of tandemly repeated genes were removed prior to analysis, as were pseudogenes, non-protein coding genes, and transcripts with fewer than 300 reads from RNA-seq in any time-point. **A:** moving average (window size 11 genes) was calculated from log_2_-regulation amplitudes across all chromosomes. The lower panel represents chromosome 1 only; dashed lines indicate borders of transcription units, as inferred from histone modifications that are characteristic of transcriptional start sites [65]. **B:** Moving averages of regulation amplitudes were calculated across 1000 genomes of randomly shuffled genes and the peak value from each was recorded. The peak value of 0.97 (an average of nearly 2-fold regulation across 11 genes) in the middle of chromosome 1 was higher than the peak value in all but four of the 1000 randomly shuffled genomes (red arrow). This peak region includes genes between Tb927.1.2290 and Tb927.1.2760. There was no other significant cluster of regulation in the genome.
(TIF)

Table S1 Read numbers attributed to *T. brucei* transcript models from the four chosen time-points after DCE-synchronization of cells. Transcript models assigned to more than 300 reads are shown. Unique reads were used when possible; for transcripts with less than 400 unique reads, multiple-mapping reads were added to the total. Read counts were normalized against the total number of reads per time-point and log_2_-transformed, and the average value for each gene across the four time-points was set to zero. Microarray data from the starve-synch experiments is also given (right of double line); log_2_-transformed expression ratios for each time-point from these were also set to an average of zero. Gene names from version 2.3 of the TriTryp database are also listed.
(XLS)

Table S2 Selected over-represented gene ontology terms found associated with transcripts that were cell-cycle regulated (amplitude thresholds as in Fig 3) and whose expression levels peaked at specific points in the DCE/RNA-seq experiment. GOstats [58] was employed using a correction for multiple testing. The lists of regulated genes used as input are given below each analysis.
(XLS)

Table S3 Categorization of genes in DNA-related processes, flagellar proteins, mitosis/cytokinesis effector proteins and RBPs using TriTrypDB text searches, proteomics studies, literature surveys or protein domain interrogation respectively.
(XLS)

Table S4 Co-regulated gene clusters, identified using K-means clustering in the TM4 software using DCE/RNA-seq data, that were used for sequence motif searches.
(XLS)

Table S5 MEME analysis of over-represented motifs in S-phase peaking transcripts. Predicted UTR sequences were extracted from genomic sequences of eight kinetoplastid species and homologues for *T. brucei* genes in co-regulated cluster #1 (Fig. 6) were collected. The best over-represented motif in cluster #1 relative to a non-regulated gene set was calculated for each species using MEME [64]. Residues in yellow or red possess >1 bit or >2 bits of information content respectively; residues in bold are non-variant in the consensus sequence generated.
(DOCX)
