# Supplementary figures and images for: The Cell Cycle Regulated Transcriptome of Trypanosoma brucei
Source: PLoS One. 2011 Mar 31;6(3):e18425. doi: 10.1371/journal.pone.0018425 (PMC3069104; doi:10.1371/journal.pone.0018425)

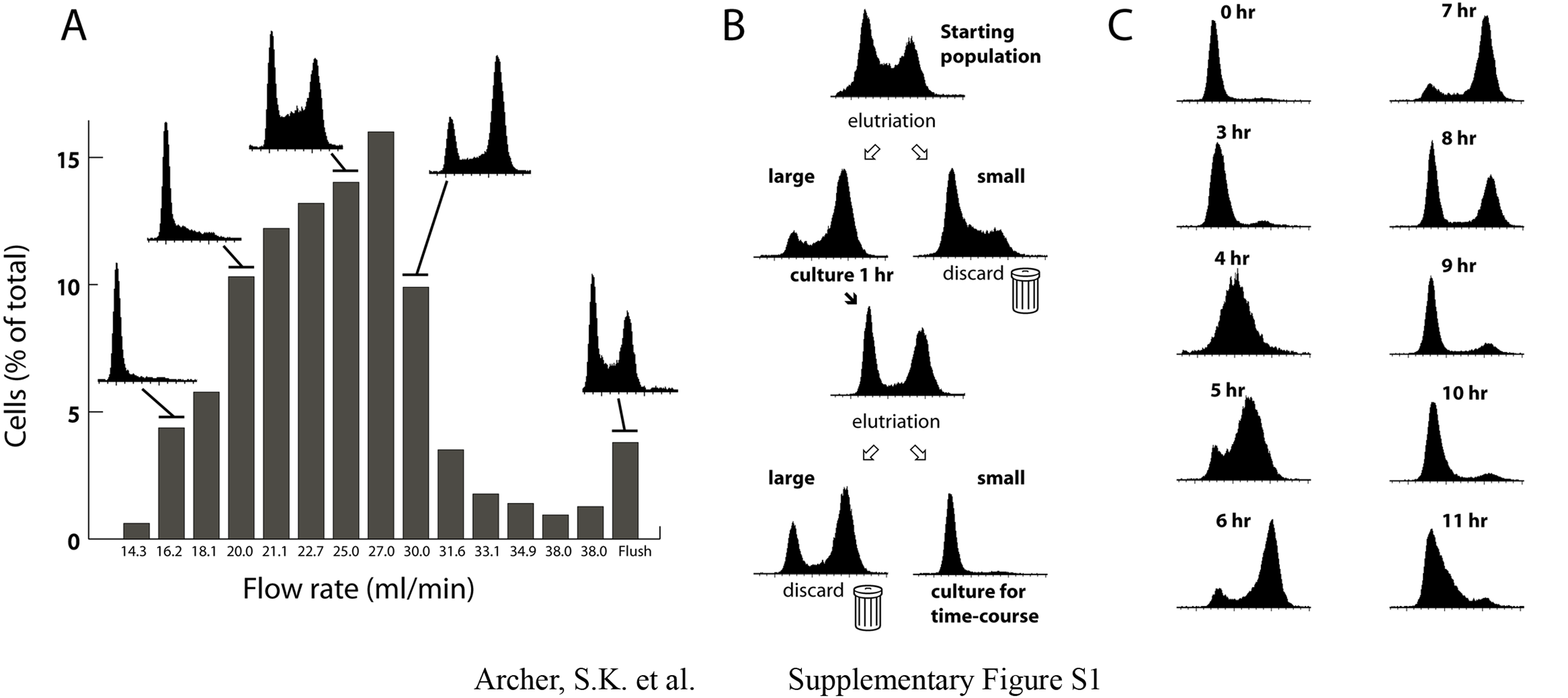

Supplement: Figure S1 — Development of a DCE procedure for isolating synchronous PC cells. A: Cell yield after fractionation of a log-phase PC culture using a constant centrifugal force (4,700×g) and increasing flow-rates. Cells from each fraction were saved for flow cytometry; results for selected fractions are shown. B: Schematic for the DCE procedure showing flow cytometry data for each step, from a pilot experiment. C: Flow cytometry results taken at various times after commencement of culturing of DCE-selected cells. (TIF) [file pone.0018425.s001.tif]

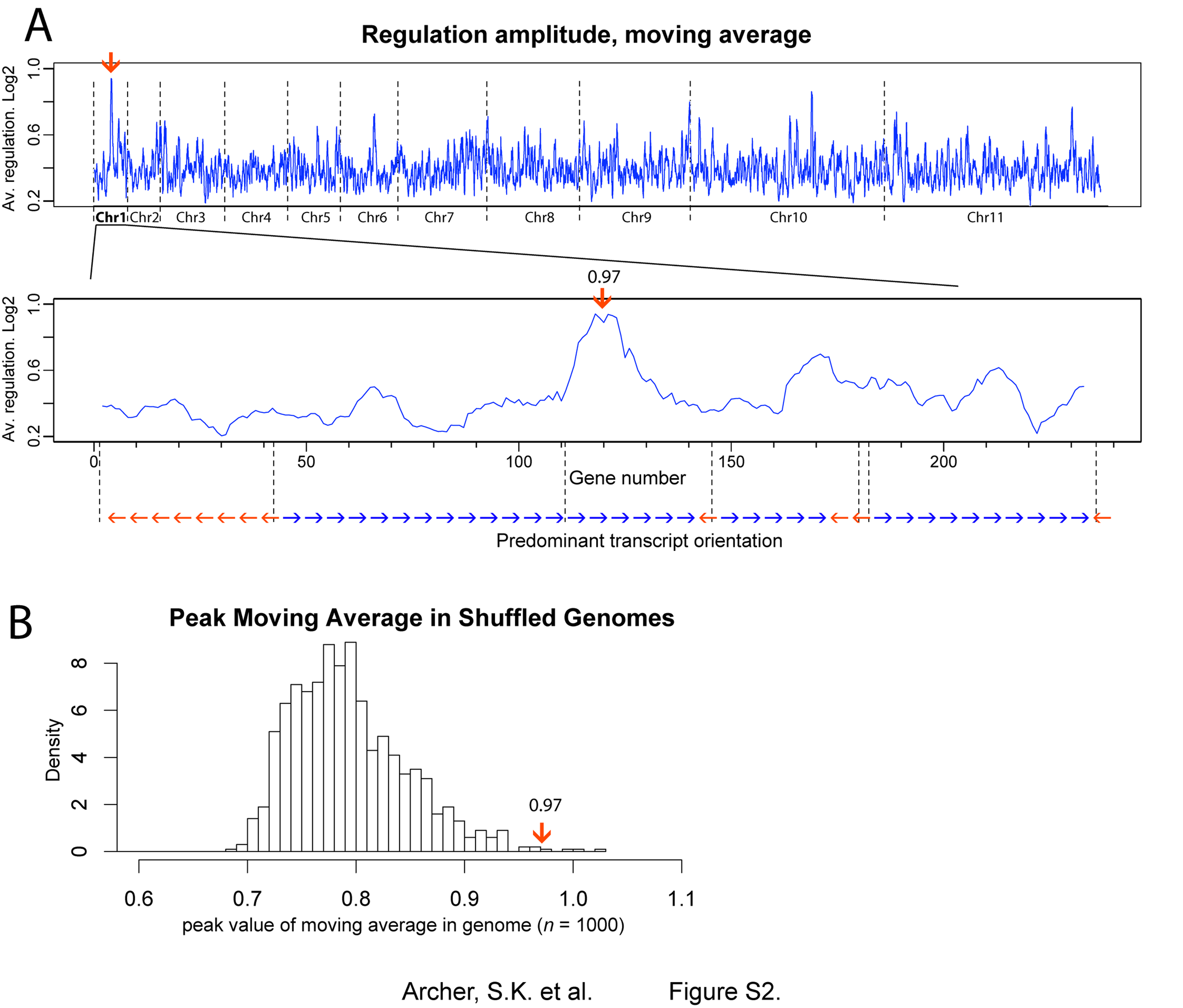

Supplement: Figure S2 — Averaging cell-cycle regulatory amplitude across genomic regions reveals only one small cluster of regulated genes. All but one copy of tandemly repeated genes were removed prior to analysis, as were pseudogenes, non-protein coding genes, and transcripts with fewer than 300 reads from RNA-seq in any time-point. A: moving average (window size 11 genes) was calculated from log2-regulation amplitudes across all chromosomes. The lower panel represents chromosome 1 only; dashed lines indicate borders of transcription units, as inferred from histone modifications that are characteristic of transcriptional start sites [65]. B: Moving averages of regulation amplitudes were calculated across 1000 genomes of randomly shuffled genes and the peak value from each was recorded. The peak value of 0.97 (an average of nearly 2-fold regulation across 11 genes) in the middle of chromosome 1 was higher than the peak value in all but four of the 1000 randomly shuffled genomes (red arrow). This peak region includes genes between Tb927.1.2290 and Tb927.1.2760. There was no other significant cluster of regulation in the genome. (TIF) [file pone.0018425.s002.tif]
