# Supplementary material for: The Cell Cycle Regulated Transcriptome of Trypanosoma brucei
Source: PLoS One. 2011 Mar 31;6(3):e18425. doi: 10.1371/journal.pone.0018425 (PMC3069104; doi:10.1371/journal.pone.0018425)
Supplement: Table S5 — MEME analysis of over-represented motifs in S-phase peaking transcripts. Predicted UTR sequences were extracted from genomic sequences of eight kinetoplastid species and homologues for T. brucei genes in co-regulated cluster #1 (Fig. 6) were collected. The best over-represented motif in cluster #1 relative to a non-regulated gene set was calculated for each species using MEME [64]. Residues in yellow or red possess >1 bit or >2 bits of information content respectively; residues in bold are non-variant in the consensus sequence generated. (DOCX) [file pone.0018425.s007.docx]

**Table S5.** MEME analysis of over-represented motifs in S-phase peaking transcripts.

| **species** | **best motif consensus** | **E value (log_10_)** | **motif occurrence/ total genes** |
| --- | --- | --- | --- |
| *T. brucei* | T**TAGAT** | +28 | 38/52 |
| *T. congolense* | **TAGAT** | +30 | 20/34 |
| *T. vivax* | **TAGA**T**T** | +40 | 16/29 |
| *T. cruzi* | TA**TAGA**T**G** | +25 | 24/33 |
| *L. major* | TTC**T**A**GA**T | +11 | 19/21 |
| *L. infantum* | **TAGAT** | +16 | 18/27 |
| *L. mexicana* | **TAGA**TCT**T** | +16 | 25/26 |
| *L. braziliensis* | T**TAGA**T**T** | +20 | 15/28 |

Predicted UTR sequences were extracted from genomic sequences of eight kinetoplastid species and homologues for *T. brucei* genes in co-regulated cluster #1 (Fig 6) were collected. The best over-represented motif in cluster #1 relative to a non-regulated gene set was calculated for each species using MEME [64]. Residues in yellow or red possess > 1 bit or > 2 bits of information content respectively; residues in bold are non-variant in the consensus sequence generated.
